# Correction: Single-cell gene expression patterns in lupus monocytes independently indicate disease activity, interferon and therapy

**DOI:** 10.1136/lupus-2016-000202corr1

**Published:** 2017-09-11

Jin Z, Fan W, Jensen MA, *et al*. Single-cell gene expression patterns in lupus monocytes independently indicate disease activity, interferon and therapy. *Lupus Sci Med* 2017;4:e000202. doi:10.1136/lupus-2016-000202

Two corrections have been made for this article.

The authors Zhongbo Jin and Wei Fan are joint first authors and contributed equally to the manuscript.

Figure 3 is not correct in the published article. The correct Figure 3 is shown below.

**Figure 3:** Hierarchical clustering of non-classical monocytes from patients and controls, with tracks indicating individuals, IFN score, SLEDAI score and prednisone usage. Each single cell forms a single row, and each column corresponds to an individual gene. All non-classical cells and genes are shown. Lower right corner inset shows heat map colour scheme key, with units indicating the delta CT values for each transcript. Colour codes for the data columns: Subject—white: healthy subjects; each colour represents one subject with SLE; interferon (IFN) score—darker green means higher IFN score; systemic lupus erythematosus disease activity index (SLEDAI) score—red: SLEDAI ≥10; pink: SLEDAI 3 to 6; white: SLEDAI 0 to 2; prednisone—dark blue: 20 mg/day; light blue: less than 10 mg/day; white: no prednisone. Black horizontal bars demarcate groups of cells that correspond to controls, high IFN, high SLEDAI and high-dose prednisone.



